# Omadacycline for community-acquired infections: A systematic review and single-arm meta-analysis

**DOI:** 10.7705/biomedica.7814

**Published:** 2026-04-28

**Authors:** Wellgner Fernandes Oliveira Amador, Cainã Araújo Saraiva, Pandora Eloá Oliveira Fonseca, Sávio Benvindo Ferreira

**Affiliations:** 1 Departamento de Medicina, Universidade Federal de Campina Grande, Cajazeiras, Brasil Universidade Federal de Campina Grande Universidade Federal de Campina Grande Cajazeiras Brasil

**Keywords:** Anti-bacterial agents, bacterial infections, community-acquired infections, treatment outcome, adverse events, mortality, antibacterianos, infecciones bacterianas, infecciones comunitarias adquiridas, resultado del tratamiento, eventos adversos, mortalidad

## Abstract

**Introduction.:**

Community-acquired infections have a significant burden on healthcare systems and are caused by a variety of pathogens with different severities. Increasing microbial resistance complicates treatment, highlighting the need for new therapies. Omadacycline, a novel antibiotic, has broad-spectrum activity against both Gram-positive and Gram-negative pathogens, particularly those involved in community-acquired infections.

**Objective.:**

To evaluate omadacycline’s efficacy and safety for treating community-acquired infections.

**Materials and methods.:**

We searched PubMed, Embase, and Cochrane for randomized and non-randomized studies about omadacycline for community-acquired infections, with no time restrictions. Primary outcomes were clinical success at the end of treatment and post-treatment evaluation. Secondary outcomes included the risk of adverse events. Statistical analysis was performed using R software.

**Results.:**

We analyzed data from 10 studies involving 1666 patients, addressing infections such as pneumonia, wound infections, cellulitis, major abscesses, lower extremity ulcers, erysipelas, cystitis, and bone or joint infections. The analysis showed a 90% (1138 of 1264 patients; 95% CI: 88 - 91) clinical success rate at the end of treatment and 84% (1288 of 1534 patients; 95% CI: 82 - 86) success rate at post-treatment evaluation. Adverse events occurred in 45% (747 of 1662; 95% CI: 32 - 59), with 18% [181 of 1008; 95% CI: 13 - 24] experiencing gastrointestinal issues. The mortality rate was 0% (0,8%) (13 patients; 95% CI: 0 - 1).

**Conclusions.:**

Omadacycline is a promising option for treating community-acquired infections.

## Resumen

**Introducción.:**

Las infecciones adquiridas en la comunidad suponen una carga considerable para los sistemas de salud y son causadas por diversos agentes patógenos que pueden presentar distintos grados de gravedad. El aumento de la resistencia microbiana complica el tratamiento, lo que destaca la necesidad de nuevas terapias. La omadaciclina, un nuevo antibiótico, tiene una actividad de amplio espectro contra agentes patógenos tanto grampositivos como gramnegativos, en particular los implicados en infecciones adquiridas en la comunidad.

**Objetivo.:**

Evaluar la eficacia y seguridad de la omadaciclina para el tratamiento de infecciones adquiridas en la comunidad.

**Materiales y métodos.:**

Se buscaron estudios aleatorizados y no aleatorizados sobre omadaciclina para infecciones adquiridas en la comunidad, en PubMed, Embase y Cochrane, sin restricciones de tiempo. Los resultados primarios fueron el éxito clínico al final del tratamiento y la evaluación posterior al tratamiento. Los resultados secundarios incluyeron el riesgo de eventos adversos. El análisis estadístico se hizo con el *software* R.

**Resultados.:**

Se analizaron datos de diez estudios que incluyeron 1666 pacientes y que abordaron infecciones como neumonía, infecciones de heridas, celulitis, abscesos importantes, úlceras de las extremidades inferiores, erisipela, cistitis e infecciones óseas o articulares. El análisis mostró una tasa de éxito clínico del 90 % (1138 de 1264 pacientes; IC_95%_: 88 - 91) al final del tratamiento y del 84 % (1288 de 1534 pacientes; IC_95%_: 82 - 86) postratamiento. Ocurrieron eventos adversos en el 45 % de los casos (747 de 1662; IC_95%_: 32 - 59), con un 18 % que experimentaron problemas gastrointestinales (181 de 1008; IC_95%_: 13 - 24). La tasa de mortalidad fue del 0 % (0,8%) (13 pacientes; IC_95%_: 0 - 1).

**Conclusión.:**

La omadaciclina es una opción prometedora para el tratamiento de las infecciones adquiridas en la comunidad.

Due to the persistent increase in antimicrobial resistance, the World Health Organization (WHO) ranked it as one of the top ten global public health threats in 2019 ^1^. In Europe alone, antibiotic-resistant infections are estimated to cause around 33 000 deaths each year ^2^. Therefore, developing new strategies to address antibiotic resistance is essential for improving healthcare and patient outcomes.

In this sense, omadacycline (Nuzyra™) is a new, first-in-class, orally active aminomethylcycline antibiotic for various infections ^3^. Developed by Paratek Pharmaceuticals, it comes in intravenous and oral forms with broad antibacterial activity ^4^. Additionally, The Food and Drug Administration (FDA) recently approved it for acute skin and skin structure infections and community- acquired bacterial pneumonia treatment, which are community-acquired infections burdening the health system and causing global mortality ^5^^,^^6^.

Omadacycline is a tetracycline antibiotic that circumvents common bacterial resistance mechanisms such as efflux pumps and ribosomal protection proteins ^3^. *In vitro* activity of omadacycline shows activity against Gram-positive aerobes-including multi-drug-resistant *Staphylococcus aureus, Streptococcus pneumoniae* and *Enterococcus* spp.- and Gramnegative aerobic bacteria such as *Legionella* spp. and *Chlamydia* spp. ^7^.

Similarly, *in vivo* experiences have demonstrated its potency against bacterial strains. In a mouse urinary tract model, omadacycline efficacy was comparable to gentamicin and better than ciprofloxacin against ciprofloxacin-resistant *Escherichia coli* strain ^8^. Another study showed that omadacycline activity was similar to *in vitro* and *in vivo* efficacies of daptomycin and vancomycin against *Enterococcus faecium* strains in a mouse peritonitis model ^9^.

Therefore, this systematic review and single-arm meta-analysis aims to evaluate clinical success at the end of treatment and at post-treatment, as well as adverse events and mortality, in patients with community-acquired infections treated with omadacycline.

## Materials and methods

This systematic review followed the recommendations of the Cochrane Collaboration and the Preferred Reporting Items for Systematic Reviews and Meta-Analysis (PRISMA) guidelines ^10^. We registered the study in the Prospective Register of Systematic Reviews (PROSPERO) under protocol number CRD42024549838.

**Figure 1.**
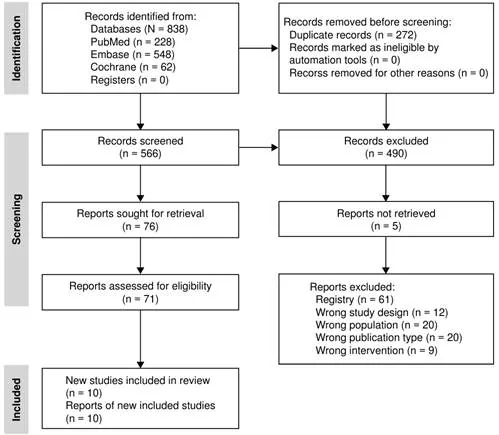
PRISMA flow diagram for study selection

An initial search identified 838 articles. After removing 272 duplicates, 566 studies were screened based on their titles and abstracts. Out of these, 490 records were excluded, leaving 76 reports for further retrieval. Ultimately, 71 studies underwent full-text evaluation, with 61 being excluded (figure 1). Therefore, this systematic review and meta-analysis included 10 studies involving a total of 1666 patients with community-acquired infections ^11^^-^^20^.

Table 1 details the baseline characteristics of the included studies. The articles were published from 2012 to 2023 and most of them (90%) were performed in the United States. This review included six randomized controlled trials, one prospective open-label trial, and three retrospective cohort articles. Multiple treatment plans were administered among patients, either intravenously or orally.

**Table 1.** Baseline characteristics of the included studies

Author, year	Sites	Type of study	Period	Treatment plan	Subjects
Ghali, 2023	16 sites in USA	Retrospective	2020.01 - 2023.3	OMC in any dosage form for ≥ 72h	75
NCT03425396	23 sites in USA	RCT	2009.04 -2012.04	OMC 300 mg (p.o.) q24h + OMC 450 mg (p.o.) q12h x Nitrofurantoin 100 mg (p.o.) q12h	171 x 54
NCT00865280	7 sites in USA	RCT	2009.04 -2010.04	OMC 100 mg (IV) q24h/300 mg (p.o.) q24h X LZD + 68 x 72 MOX 600 mg (IV) q12h/600 mg (p.o.) q12h	68 x 72
Noel, 2012	11 sites in USA	RCT	2016.08 -2017.06	OMC 100 (IV) q24h/200 mg (p.o.) q24h x LZD 600 mg (IV) q12h/600 mg (p.o.) q12h	118x116
OASIS-1,2019	55 sites in USA, Perú, South Africa and Europe	RCT	2015.06 - 2016.05	OMC 100 mg (IV) q12h for 1 day+ 100 mg (IV) q24h for 2 days+100 mg (IV) or 300 mg (p.o.) q24h x LZD 600 mg (IV) q12h for 3 days + 600 mg (IV) or 600 mg (p.o.) q12h	316x311
OASIS-2, 2019	33 sites in USA	RCT	2016.08 -2017.06	OMC 450 mg (p.o.) q24h for 2 days + 300 mg (p.o.) q24h x LZD 600 mg (p.o.) q12h for 2 days + 600 mg (p.o.) q12h	368 x 367
Overcash, 2019	3 sites in USA	Prospective open-label	2016.05- 2016.09	OMC 200 mg (IV) q24h/300 mg (p.o.) q24h x OMC 300 mg (p.o.) a12h/300 mg (po) q24h x OMC 450 mg (p.o.) q12h/450 mg (p.o.) q24h	31
Stets, 2019	86 sites in Europe, North America, South America, the Middle East, Africa, and Asia	RCT	2015.11 -2017.02	OMC 100 mg (IV) ql2h/100 mg (IV) q24h x MOX 400 mg (IV) q24h	386 x 388
Wang, 2023	China	Retrospective	2021.09 - 2022.10	OMC (dosage not specified)	16

RCT: randomized controlled trial; OMC: omadacycline; LZD: linezolid; MOX: moxifloxacin; IV: intravenous; p.o.: oral; q12h: every 12 hours; q24h: every 24 hours; NA: not available

For statistical analysis at the end of treatment, 1264 patients comprised the study population, given that only five studies presented this outcome data. For post-treatment evaluation, nine studies were analyzed, involving a total of 1534 individuals. In the safety analysis of the studies 1662 patients were included.

The community-acquired infections to which omadacycline was administered were mainly pulmonary (reported in 40% of the studies) and skin and soft tissue diseases (80% of the studies), such as wound infection, cellulitis, major abscess, lower extremity ulcer, and erysipelas. Additionally, cases of cystitis and bone or joint infection also comprised the study population ^15^^,^^16^^,^^21^. The studies demonstrated a variety of Gram-positive and Gram-negative pathogens at baseline.

### Search strategy

A systematic search on PubMed (MEDLINE), Embase, and Cochrane and Clinical Trial databases was conducted on May 17, 2024. In addition, the references of included articles and prior systematic reviews and metaanalyses were evaluated for additional studies. The following search strategy was used: (“Omadacycline” OR “Nuzyra” OR “Amadacycline” OR “PTK 0796”) AND (“Community-acquired infections” OR “Infections” OR “Bacterial infection” OR “Bacterial diseases” OR “Infection, bacterial” OR “Skin Disease, Infectious” OR ABSSSI OR “Infectious Skin Diseases” OR “Pneumonia” OR CABP OR “Respiratory Tract Infections”).

### Inclusion criteria

The eligible studies for this systematic review were:

1. original studies evaluating either efficacy or safety without time restriction,
2. papers including patients of any age with community-acquired bacterial infections,
3. studies in which patients were being treated with omadacycline in any dosage, regardless of whether there was a comparative group,
4. studies reporting clinical outcomes, such as clinical success and adverse events,
5. studies that conduct tests with patients.

We excluded studies characterized by:

1. overlapping populations,
2. populations of no interest,
3. republished literature,
4. protocols without results,
5. reviews, abstracts, case reports, case series, background articles, expert opinion letters, *in vivo* and *in vitro* studies, etc.,
6. data from the same clinical trial.

No language restrictions were applied during the search process.

Baseline characteristics of infections treated with omadacycline are described in table 2.

**Table 2.** Baseline characteristics of infections treated with omadacycline*

Author, year	Infection site (n, %)	Pathogens detected at baseline (n, %)
Ghali, 2023	NTM pulmonary disease (33, 44.0), Skin and soft tissue disease (17, 27.4) Osteomyelitis (10, 16.1) Invasive prosthetic device (5, 6.7) Intra-abdominal (3, 4) Other** (5, 6.7)	*Mycobacterium abscessus* (60, 80) *Mycobacterium chelonae* (9, 12) *Mycobacterium avium complex* (7, 9.3) *Mycobacterium fortuitum* (4, 5.3)
Mingora, 2023 NCT03425396	Pulmonary (94, 80) Skin and soft tissue (12, 10) Multiples sites of infection (6, 5) Peritonitis (3, 3) Disseminated (1,1) Bone or joint (1,1) Cystitis (225, 100%)	*Subspecies of Mycobacterium abscessus* (117, 100) *Mycobacterium abscessus* (58, 49.6) *Mycobacterium massiliense* (11,9.4) *Mycobacterium bolletii* (2, 1.7) Not identified (46, 39.3)
NCT00865280	Complicates skin and skin structure infection (143, 100) Wound infection (26, 18.2) Cellulitis (84, 58.7) Major abscess (33, 23)	
Noel, 2012	Major abscess (73, 66) X (72, 67) Wound infection (21, 19) X (17, 16) Lower extremity ulcer (9, 8) X (9, 8) Cellulitis (8, 7) X (10, 9)	*Staphylococcus aureus* (72, 61) x (55, 47.4) MRSA (44, 37.3) x (32, 27.6) Gram-positive bacteria other than S. aureus (3, 2.5) x (7, 6) Gram-negative bacteria (2, 1.7) x (1,0.8)
OASIS-1,2019	Wound infection (102, 32.3) X (104, 33.4) Cellulitis or erysipelas (123, 38.9) X (118, 37.9) Major abscess (91,38.8) X (89, 28.6)	*Staphylococcus aureus* (156, 49.4) x (151,46.6) *Methicillin-susceptible* (88, 27.8) x (102, 32.8) *Methicillin-resistant* (69, 21.8) x (50, 15.5) *Streptococcus anginosus group* (47, 14.9) x (37, 11.9) *Streptococcus constellatus* (25, 7.9) x (14, 4.5) *Streptococcus intermedius* (12, 3.8) x (18, 5.8) *Streptococcus pyogenes* (11,3.5) x (18, 5.8) *Enterococcus faecalis* (10, 3.2) x (13, 4.2)
OASIS-2, 2019	Wound infection (210, 58 X 214, 59) Cellulitis or erysipelas (86, 24 X 84, 23) Major abscess (64, 18 X 62, 17)	Gram-positive aerobes (270, 98) x (278, 97) *Staphylococcus aureus* (220, 80) x (233, 81) MSSA (120, 44) x (130, 45) MRSA (104, 38) x (107, 37) *Streptococcus pyogenes* (29, 11) x (16, 6) *Streptococcus anginosus group* (57, 21) x (45, 16) *Enterococcus faecalis* (7, 3) x (10, 4)
Overcash, 2019	Cystitis (31, 100)	Gram-negative pathogens (16, 88.9) *Escherichia coli* (10,55.6) *Klebsiella pneumoniae* (3, 16.7) *Proteus mirabilis* (4, 22.2) Gram-positive pathogens (4, 22.2) *Aerococcus urinae* (1, 5.6) *Enterococcus faecalis* (1, 5.6) Staphylococcus saprophyticus (1, 5.6) *Streptococcus agalactiae* (Group B) (1,5.6)
Stets, 2019	CBAP (774, 100)	Gram-positive aerobic bacteria (61, 15.8) x (56, 14.4) *Streptococcus pneumoniae* (43, 11.1) x (34, 8.7) *Penicillin-susceptible* (26, 6.7) X (22, 5.7) Macrolide-resistant (10, 2.6) X (5, 1.3) Tetracycline-resistant (16, 4.1) X (17, 4.4) *Staphylococcus aureus* (11,2.8) X (11,2.8)
Wang, 2023	Pulmonary (16, 10)	*Chlamydia psittaci* (16, 100)

NTM: nontuberculous mycobacteria

* Data are presented as number of cases (percentage)

** Other infection sources were as follows: otitis media (n = 2), infective endocarditis (n = 1), central line (n = 1), external

### Data extraction

After removing duplicates, two out of the authors screened the titles and abstracts, independently evaluating the full-text articles for inclusion based on pre-specified criteria. Discrepancies were resolved by consensus with a third author. Data extraction was conducted prioritizing information relevant to the study’s objective and focusing on observational and randomized clinical trials.

### Quality assessment

The risk of bias for randomized controlled trials was assessed using the Cochrane Risk of Bias 2 (RoB 2) tool ^21^. The evaluation of bias was performed by two independent reviewers. The RoB 2.0 tool rates the risk of bias as either high, some concerns, or low across five domains: selection, performance, detection, attrition, and reporting biases.

For non-randomized studies, the ROBINS-I ^22^ tool was used. In case of discordant results, a third reviewer made the final decision after a discussion with the team. Risk-of-bias plots were generated using Robvis ^23^.

### Definitions and outcome measurement

The intent-to-treat population comprised all patients who received omadacycline, regardless of the microbiological profile. Clinical success, defined as “survival, investigator-assessed symptom improvement, without receipt of rescue antibacterial therapy”, was evaluated at the end of treatment, 2 or 3 days after the last dose of omadacycline; and at posttreatment, 14 to 90 days after the last dose of the antibiotic.

Adverse events were defined as “any events that arose during or after treatment, including mild and serious adverse events”. Gastrointestinal events included both nausea and vomiting outcomes. Separately, mortality linked exclusively to deaths due to infection or omadacycline failure was assessed.

### Statistical analysis

This single-arm proportion meta-analysis investigates the use, efficacy and safety of omadacycline on community-acquired infections. Statistical analysis was carried out using R software and RStudio™, version 2024.04.1+748 (R Core Team, Vienna, Austria).

Random-effects modeling was used for analysis, assuming that the data come from varied populations with different distributions and individual profiles. DerSimonian and Laird’s random-effects model was employed to calculate the combined proportion of each adverse event. Aditionally, a 95% confidence interval was taken into account. The results were presented as pooled analysis in forest plots.

In addition, we adopted the Cochrane Q test, *X* ^2^ test and I² statistic to examine heterogeneity across studies; p <0.10 and I^2^ 30% were considered significant for heterogeneity.

Publication bias was assessed through visual inspection of funnel plots based on point estimates and study weights, as well as by using a regression test for funnel plot asymmetry (Egger’s test).

## Results

### Efficacy

**Figure 2. A):**
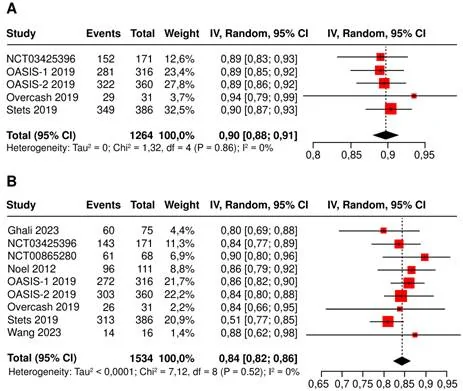
Clinical success at the end of treatment; **B)** clinical success at post-treatment evaluation

Figure 2A shows the forest plot for the overall incidence of clinical success evaluated at the end of treatment. A pooled analysis revealed an overall end of treatment clinical success incidence rate of 90% (95% CI: 88 - 91; I^2^ = 0%).

Figure 2B reveals an overall post-treatment evaluation clinical success rate of 84% (95% CI: 82 - 86; I^2^ = 0%).

Both outcomes demonstrated a low heterogeneity (p = 0.86,1^2^ = 0%; p = 0,52,1^2^ = 0%, respectively).

### Safety

**Figure 3. A):**
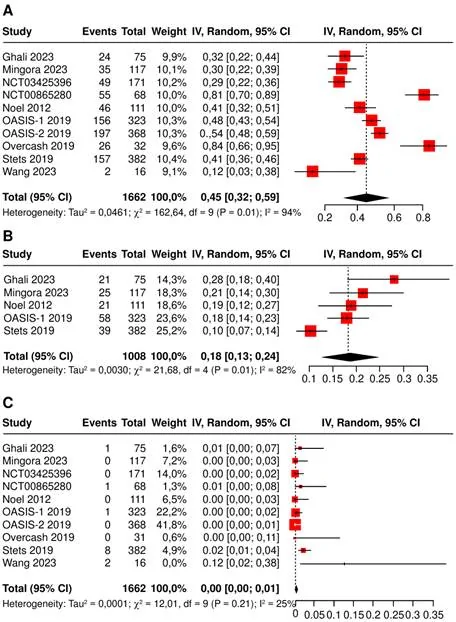
Adverse events during the treatment with omadacycline; **B)** gastrointestinal events; **C)** mortality rate attributed to omadacycline

CI: Confidence interval; IV: intravenous

Among the 1662 patients included in the safety analysis of the studies, 747 cases (45%) of adverse events directly attributed to omadacycline were recorded. The pooled analysis confirms an overall incidence rate of adverse events of 45% (95% CI: 32 - 59; I² = 94%) (figure 3A).

Most studies report a high prevalence of gastrointestinal events, such as nausea and vomiting, which were mild and transient and, in most cases, did not lead to omadacycline discontinuation. Hence, given that gastrointestinal numbers overlapped in some studies, a pooled analysis of five studies was performed (N = 1008) (figure 3B), which revealed a gastrointestinal adverse events incidence rate of 18% (95% CI: 13 - 24; I² = 82%) (14,18-22). Only one trial reported treatment discontinuation of 23 patients due to adverse events ^15^.

Additionally, the overall mortality incidence rate directly related to omadacycline failure or infection complication is shown in figure 3C. A pooled analysis demonstrated an overall mortality incidence rate of 0% (95% CI: 0 -1; I^2^ = 25%). In this context, two studies reported deaths due to comorbid conditions that were not infection-related, which were not considered in composing the statistics ^14^^,^^15^. Another one affirmed that the cause of death was possibly attributed to secondary infections of the patient ^23^.

High heterogeneity was detected among studies for adverse events (p < 0.01, I^2^= 94%), gastrointestinal adverse events (p < 0,01,1^2^ = 82%) and mortality rates (p = 0.21,1^2^ = 25%), which may be attributed to selection bias in the non-randomized small-sized studies and different dosages and administration formulations of omadacycline.

### Quality assessment

Most published studies were categorized as having a low or moderate risk of bias (appendix 1). Three randomized controlled trials were considered at low risk of bias, two were classified as some concerns and one had a high risk of bias due to open-label characteristics. The risk of bias for non- randomized studies was classified as moderate for two included studies and low for one article.

Considering the results, to assess the level of heterogeneity in our study, a leave-one-out sensitivity test was carried out (appendix 2) by systematically removing each study from the pooled estimates of the primary outcomes. It did not substantially alter the results for either end of treatment, posttreatment evaluation clinical success, adverse events, gastrointestinal events, or mortality outcomes.

Moreover, the visual inspection of the funnel plot with studies for each outcome assessed suggests a subtle asymmetry toward the left side (appendix 3). Hence, an Egger’s regression-based test was conducted and showed significance only for the mortality and gastrointestinal adverse events rates, indicating likely evidence for publication bias.

Then, it was hypothesized that this asymmetry might be related to:

1. the fact that not all studies were randomized;
2. several diseases with different gravity levels were being treated;
3. individual characteristics -such as comorbidities-were not taken into account; so, parallel conditions could have influenced death outcomes in some studies;
4. the different doses of omadacycline among trials -Tor example, in Stets *et al*. ^22^, patients were treated with a low dose of 100 mg twice daily initially, while in O’Riordan *et al*. ^20^ a high daily dose of 450 mg was administered, which might have yielded a higher bioavailability of omadacycline and less negative outcomes-;
5. the different formulations of omadacycline; for example, oral administrations led to a major number of gastrointestinal events when compared to intravenous administration.

Therefore, in future trials, if clinicians assign individuals to fixed ideal doses randomly, adverse events and mortality rates may exhibit greater uniformity across studies.

## Discussion

This systematic review and single-arm meta-analysis comprehensively analyzed omadacycline efficacy and safety profiles in treating community- acquired infections. Data from ten studies included with 1666 patients and a focused assessment of two significant endpoints was conducted: clinical success and adverse events associated with omadacycline use.

The main findings were the overall incidence of clinical success at both end of treatment and post-treatment evaluation was 90 and 84%, respectively. Moreover, the adverse events incidence rate was 45%, of which the majority included mild events, such as gastrointestinal outcomes (18%). No infection-related mortality was observed. In this context, previous meta-analyses aimed to evaluate omadacycline action ^24^^-^^26^.

Nonetheless, this study is relevant for several reasons. First, it uses rigorous inclusion criteria and advanced statistical techniques that reduce bias and increase the accuracy of results. In addition, it includes more recent studies, providing an up-to-date data view that reflects the most current practices and data. Finally, the detailed and transparent approach increases confidence in the validity of the findings and contributes to the reproducibility of the research.

Community-acquired diseases are those contracted outside healthcare settings, such as in homes, schools, and workspaces, including illnesses like flu, pneumonia, and urinary infections ^27^. Therefore, the main challenge for the healthcare system is to diagnose and treat these infections quickly, dealing with pathogen variability and antibiotic resistance. Additionally, the rapid spread can lead to outbreaks, requiring efficient responses and prevention strategies to control transmission. In this context, omadacycline, a novel antibiotic recently approved by the FDA, proves useful as a treatment target for these diseases.

Concerning investigator-assessed clinical response or success, end of treatment endpoint incidence ranged from 93 to 88% in the included studies, while post-treatment evaluation outcome incidence ranged from 90 to 80%. In this sense, this data is consistent with other trials involving patients with acute bacterial infections, with end of treatment clinical success varying from 81.6 to 79.4%, which suggests that investigator-assessed early treatment success had a high positive predictive value for late clinical cure ^28^^,^^29^. This means that, since omadacycline has an oral formulation, it allows for step- down therapy from the intravenous formulation, representing a clinical tool for earlier hospital discharge, outpatient therapy, and cost savings ^3^.

Adverse events occurred in all trials, with an incidence rate ranging from 12 to 83%. Hereupon, gastrointestinal events, such as nausea and vomiting, comprised 18% of patients in the included studies and were the most reported adverse events. In this sense, a notable difference in nausea and vomiting events between studies that administered omadacycline orally ^20^ (173 patients, 47%) and intravenously ^19^ (57 patients, 17.7%) may suggest that oral omadacycline is likely to cause gastrointestinal irritation, increasing gastrointestinal adverse events ^20^^,^^19^.

Furthermore, infection-related mortality incidence was null. Six studies reported all-cause mortality, but only five of them revealed deaths linked to infection and not just comorbidities-related complications. In this regard, the mortality rates were similar between omadacycline and other comparative drugs, such as moxifloxacin (2.1% to 1%) ^22^ and linezolid (odds ratio (OR) = 0.76; 95% Cl: 0.17 to 3.40; p = 0.72) ^25^, which was not statistically significant ^22^^,^^25^.

Nevertheless, the present study has some limitations. It included non- randomized, small-sized, and early-phase trials, which could introduce selection bias to the review. However, considering the methodological quality of the included studies and the small number of randomized controlled trials published, these smaller trials have contributed greatly to the meta-analysis. It was unable to perform subgroup analysis to assess each condition separately because individualized patient data was not given in all studies. Most patients were previously treated with other antibiotics, which may confound if the outcomes investigated were really due to omadacycline or to other drugs. Several kinds of community-acquired infections were combined, which have diverse microbiologic causes and treatment guidelines.

Future studies should aim to further clarify the role of omadacycline in specific patient populations, such as those with comorbidities, immunosuppression, or severe infections. Additionally, randomized controlled trials with direct comparisons to other first-line antibiotics are needed to better position omadacycline in clinical guidelines.

Long-term pharmacovigilance and surveillance programs are also crucial to monitor the development of resistance of each pathogen and assess the safety profile in real-world settings. Standardizing dosing regimens and administration routes across various infection types will contribute to optimizing clinical outcomes and minimizing adverse effects.

In conclusion, this comprehensive systematic review and meta-analysis supports the efficacy and safety of omadacycline for community-acquired infections. The overall incidence of clinical success assessed at the end of treatment and post-treatment was high.

Although adverse events occurred in 45% of the population, the mortality rate was null and most of the cases were mild and did not lead to omadacycline discontinuation. Based on this, further research is needed to help to enhance our understanding of omadacycline in diverse types of infections and patient populations.
